# The role of attachment insecurity in the emergence of anxiety symptoms in children and adolescents with migraine: an empirical study

**DOI:** 10.1186/s10194-017-0769-3

**Published:** 2017-05-30

**Authors:** Riccardo Williams, Luigi Leone, Noemi Faedda, Giulia Natalucci, Benedetta Bellini, Elisa Salvi, Paola Verdecchia, Rita Cerutti, Marco Arruda, Vincenzo Guidetti

**Affiliations:** 1 Department of Dynamic and Clinical Psychology, Sapienza University of Rome, Rome, Italy; 2 Department of Social and Developmental Psychology, Sapienza University of Rome, Rome, Italy; 3 Phd program in Behavioural Neuroscience, Department of Paediatrics and Child and Adolescent Neuropsychiatry, Sapienza University of Rome, Rome, Italy; 4 Department of Paediatrics and Child and Adolescent Neuropsychiatry, Sapienza University of Rome, Via dei Sabelli 108, 00185 Rome, Italy; 5 Glia Institute, Ribeirão Preto, São Paulo, Brazil

**Keywords:** Migraine, Anxiety, Attachment, Developmental age, Perception of security

## Abstract

**Background:**

It is widely recognised that there are associations between headache, psychiatric comorbidity and attachment insecurity in both adults and children. The aims of this study were: 1) to compare perceived attachment security and anxiety in children and adolescents with migraine without aura and a healthy control group; 2) to test whether the child’s perceived security of attachment to the mother and the father mediated the association between migraine and anxiety.

**Methods:**

One hundred children and adolescents with Migraine without Aura were compared with a control group of 100 children without headache. The Security Scale (measures perceived security of attachments) and the Self-Administered Psychiatric Scales for Children and Adolescents, a measure of anxiety symptoms, were administered to all participants.

**Results:**

The clinical group had lower attachment security than the control group and higher scores on all anxiety scales. Anxiety was negatively correlated with attachment. Children’s attachment to their mother mediated the increase in global anxiety in the clinical group. Insecure paternal attachment was associated with greater insecurity in maternal attachment, suggesting that there is a complex pathway from migraine to anxiety symptoms mediated by perceived insecurity of paternal attachment and hence also by perceived insecurity of maternal attachment.

**Conclusion:**

These results suggest that insecure parental attachment may exacerbate anxiety in children and adolescents with migraine and point to the importance of multimodal interventions, perhaps taking account of family relationships, for children and adolescents with migraine.

## Background

The prevalence of migraine in children and adolescents is about 7.7–9.1% [1]. It is widely recognised that headache, in particular migraine, is associated with psychiatric comorbidity in both adults and children [2–4]. The most frequently described psychiatric comorbidities are: anxiety disorders and scholastic anxiety [2], mood disorders [5], sleep disorders [6] and eating disorders [7]. However, some recent studies [8, 9] have called into question the relationship between migraine and psychiatric disorders, especially in children and adolescents with migraine. Given this controversy and its possible implications for treatment and prognosis, it is important to establish the extent of psychiatric problems in patients with migraine, and to understand their origin.

Attachment is defined as a behavioural and cognitive system that regulates an individual’s sense of internal security. In infancy, attachment security has been defined as a child’s confidence that his or her caregiver will be available and responsive in times of distress [10]. A secure pattern of attachment in childhood with an adult caregiver is a central element in the healthy development, to build self- efficacy and a good level of self-esteem, to protect the individual against stressful events and develop the foundations for mature relationships [11]. On the contrary, insecure models of attachment have to be considered as an important factor of vulnerability for psychopathology when other important stressful events and risk factors occur. In particular, attachment theorists argue that the formation of attachments is the key developmental process with regard to regulation of anxiety. Empirical research has shown that attachment insecurity in childhood and adolescence is an important correlate of anxiety disorder diagnoses, regardless of parental anxiety and psychopathological diagnoses [12]. Moreover, recent research has shown that insecure attachment may exacerbate the frequency and intensity of headache attacks, as well as contributing to associated psychopathology [13].

Given that early attachment is relevant to psychopathology and anxiety regulation during development, this pattern of findings appears consistent with the notion that children’s perceived attachment security mediates the emergence of higher anxiety levels in children and adolescents with MoA. Mediation appears a fruitful conceptual and empirical tool to investigate the hypothesized mechanism that transmits an association between an independent variable and a dependent variable (X → Y), introducing a third variable (M) - the mediator [14]. The mediation pattern would thus be represented by: X → M → Y. In our case, the mediation we anticipated would take the form: MoA → attachment security → anxiety. The empirical tenability of such an hypothesis would help further efforts to understand the mechanisms underpinning the association of migraine with anxiety.

Some findings appear consistent with such a mediation. Previous research [13] suggests that migraine has a negative effect on children’s perception of the security of their attachment relationships, and that – in turn – perceived attachment insecurity is related to anxiety. This pattern of associations suggests that attachment insecurity partially mediates the association between migraine and anxiety. To the best of our knowledge, no previous study has specifically investigated the role of children’s attachment insecurity as a potential mediator of the relationship between anxiety and migraine in children and adolescents. Our aim was to determine whether there was empirical evidence for such a mediation pattern.

We were also interested in investigating whether there were differences in how the perceived security of maternal and paternal attachments affected the migraine-anxiety association. Some evidence from longitudinal research suggests that there are distinct models of attachment for the mother and the father, respectively, and that such distinct models do not coalesce into a single model of attachment until late adolescence [15]. This implies that in an investigation of how attachment security influences anxiety in a paediatric population of migraine sufferers it is important to distinguish between maternal and paternal attachment.

To summarise, the aims of this study were: 1) to compare perceived attachment security and anxiety in a group of children and adolescents with MoA and a group of healthy controls; 2) to test whether children’s perceptions of the security of their maternal and paternal attachments mediated the migraine → anxiety association.

## Method

### Sample and procedure

We investigated these two questions via a case-control study. The clinical group was recruited consecutively from admissions to the Paediatric Headache Centre of Department of Paediatrics and Child and Adolescent Neuropsychiatry, “Sapienza” University of Rome, Policlinico Umberto I. All subjects were recruited in the period between January and December 2011 and met the following inclusion criteria: (a) Diagnosis of MoA (1.1) according to the ICHD–III criteria [16]; (b) aged between 8 and 18 years old; (c) had experienced headaches for at least 6 months; and (d) a minimum of 2 attacks per month over the last 3 months. This latter criterion was adopted in order to avoid having a heterogeneous clinical group containing patients with both sporadic and very frequent migraine episodes.

The exclusion criteria were: (a) diagnosis of secondary headache; (b) diagnosis of other types of primary headache; (b) presence of medical or psychiatric comorbidities (e.g., coeliac disease, epilepsy, diabetes, attention deficit hyperactivity disorder, depression), as these conditions could affect attachment and anxiety. The control group was instead recruited from primary and secondary schools in the province of Rome. All subjects came from the same urban area, were of Caucasian origin and from families with middle class socio-economic status (Class 2: household income = €28,000–55,000 p.a.; Class 3: household income = €55,000–75.000 p.a.; current Italian economic parameters). Both the subjects and their parents were required to give active, informed consent to participation. The study was conducted according to the criteria set out in the Declaration of Helsinki [17].

The size of the groups was sufficient to detect group differences in means of medium magnitude (Cohen’s *d* ≥ .5) with a power of .80. This level of power appeared adequate given the magnitude of anxiety scores differences between clinical and control groups generally reported in the literature on migraine [9]. As for the association between migraine and attachment security, the sample size guaranteed a power of .80 for correlations as small as 0.2, which also appeared a reasonable minimum, given the variables involved [13, 18–20].

### Measures

Anxiety was measured using the Self-Administered Psychiatric Scales for Children and Adolescents (SAFA) battery [21–24].

Perceived attachment security was evaluated using the Italian translation (Calvo V. (1998) Italian translation of the Security Scale by Kerns, Klepac e Cole, Unpublished Manuscript; Calvo V. (2008a) Description and evaluation form of the Security Scale], Unpublished Manuscript; Calvo V. (2008b) Aspects of validation of the Security Scale in Italy: internal consistency and scores distribution. Unpublished Manuscript) of the Security Scale [25]. The *SAFA* [22] is a self-report scale for children and adolescents that assesses six types of psychopathology (anxiety, depression, somatisation, obsessive-compulsive symptoms, psychogenic eating disorders and specific phobias). The psychometric properties of the battery are satisfactory: it has good internal consistency (α = .80), acceptable test-retest stability and satisfactory convergent and discriminant validity [24]. For the purposes of this study we only used the anxiety disorders scale (SAFA-A) which assesses symptoms of generalised anxiety disorder (GAD), social anxiety disorder (SAD), separation (from parents) and loss anxiety (SLA), and scholastic anxiety (SA). The total scale score provides global measure of anxiety.

The *Security Scale* is a 15-item self-report scale used to measure children and adolescents’ perceptions of the security within th relationships with their father and mother. The notion of perceived attachment security refers to the degree in which the children or adolescents feel and believe their parents are able to detect and effectively regulate their states of distress [26]. The Security scale is thus composed of two separate scales that measure the level of perceived attachment security with each parent (maternal and paternal security scales), as evaluated by the child and adolescent. A third scale is then reckoned (total scale of perceived security) by cumulating the scores of maternal and paternal scales. The reliability and validity of the Security Scale have been assessed in both child and adolescent samples [26] and it has shown satisfactory stability and internal consistency (maternal attachment: α = .64–.90; paternal attachment: α = .81–.88). The Security Scale has shown convergence with other middle-childhood measures of attachment as well as with observations of children’s interactions with their parents [25].

### Statistical analyses

**Fig. 1:**
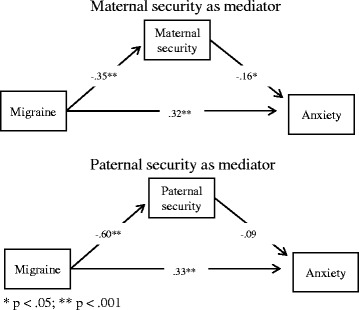
Maternal security as mediator (*upper panel*), Paternal security as mediator (*lower panel*)

We used independent-samples *t*-tests to assess differences between the clinical and control groups. Effect sizes were computed in terms of Cohen’s *d* [27], which scales mean differences relative to the pooled standard deviation of the two groups. We also anticipated a mediation pattern. Mediation represents a chain of relations where an antecedent variable relates to a mediating variable, which in turn is associated with an outcome variable [28]. The models in Fig. 1 depict the basic mediation pattern we tested. The mediation hypotheses showed in Fig. 1 were tested using the SPSS PROCESS macro (Hayes, 2013), a procedure implemented under the SPSS statistical package to compute and test mediation effects. Results of the mediation analysis allow to partition the total association of Migraine with Anxiety into an indirect association channeled by attachment (the mediator), and a direct association of migraine with anxiety that is not channeled by attachment.

## Results

Table 1 reports correlations and descriptive statistics for the pooled groups.

**Table 1:** Correlations, means and standard deviations for the variables in the study (*N* = 200)

	Group	Gender	Age	TA	GAD	SAD	SLA	SA	PSM	PSF
Group	1	-	-	-	-	-	-	-	-	-
Sex	−.030	1	-	-	-	-	-	-	-	-
Gender	.000	0	1	-	-	-	-	-	-	-
TA	.371**	.016	.131	1	-	-	-	-	-	-
GAD	.337**	−.004	.12	.811**	1	-	-	-	-	-
SAD	.372**	.023	.031	.756**	.521**	1	-	-	-	-
SLA	.240**	.083	−.003	.677**	.443**	.411**	1	-	-	-
SA	.309**	.004	.104	.762**	.560**	.490**	.380**	1	-	-
PSM	−.330**	−.135	−.009	−.259**	−.226**	−.218**	−.099	−.284**	1	-
-PSF	−.600**	−.102	.042	−.281**	−.265**	−.362**	−.116	−.248**	.372**	1
M(SD)	-	-	10.97(2.17)	44.42 (9.62)	44.69 (9.50)	46.15 (8.87)	43.05 (9.31)	48.12 (9.46)	47.41 (9.22)	47.66 (10.26)

Legend: Group: Control Group(0); Clinical Group (1); Gender: Female(0); Male (1); Total anxiety (TA), Generalized Anxiety Disorder (GAD), Social Anxiety Disorder (SAD), Separation and Loss Anxiety; (SLA), Scholastic Anxiety (SA); Security Perception toward the Mather (PSM); Security Perception toward the Father (PSF)

** *p* < 0.01 (two-tailed)

The clinical migraine group was composed of 100 children and adolescents (48 boys; 52 girls) with a mean age of 10.64 (*SD* = 2.85 years), instead the control group was composed of 100 children and adolescents (49 girls; 51 boys), with a mean age of 10.16 years (*SD* = 2.01).

The two groups had similar age (*t* < 1, *ns*) and gender distributions (χ^2^_(1)_ = 0.18, *p* = .67).

### Mean differences across groups

Table 2 presents group means for anxiety and attachment and the *t*-statistic for each comparison. Because we tested six comparisons, we set the maximum acceptable type I error rate for each *t*-test at .008 (.05/8) in order to bring the acceptable overall type I error rate close to the nominal .05 level. As Table 2 shows, all group differences were significant using this conservative criterion.

**Table 2:** Mean differences between clinical and control groups on anxiety and attachment security measures

Variable	Clinical Group Mean (SD)	Control Group Mean (SD)	t(df)	p (2-tailed)
GAD	47.92 (9.65)	41.53 (8.26)	5.00 *(196)*	<.0001
SA	49.48 (10.32)	42.89 (5.53)	5.61 *(196)*	<.0001
SLA	45.31 (8.32)	40.85 (9.76)	3.46 *(196)*	<.001
SAD	51.07 (9.93)	45.24 (8.04)	4.54 *(196)*	<.0001
PSM	44.31 (7.18)	50.39 (9.98)	*4.87 (194)*	<.0001
PSF	41.36 (7.77)	53.66 (8.64)	10.43 (193)	<.0001

Legend: Generalized Anxiety Disorder (GAD), Social Anxiety Disorder (SAD), Separation and Loss Anxiety (SLA), Scholastic Anxiety (SA); Security Perception toward the Mather (PSM); Security Perception toward the Father (PSF)

An inspection of the figures in Table 2 reveals that there were remarkable group differences on all SAFA-A subscales. For all scales the clinical group showed higher anxiety scores compared with the control group, and the differences were all highly significant (all *p*s < .001). To better gauge the size of the reported mean-differences we relied on Cohen’s *d* [27]. By this metric, values about .5 indicate medium effects, and values above .8 suggest large effects [27]. Medium-to-large effects were found for GAD (*d* = .71), SA (*d* = .80), SLA (*d* = .49) and SAD (*d* = .65) scores. Therefore one might conclude that the differences in anxiety across groups were considerable. As for the attachment scores, the clinical group showed lower levels of security for both Mother and Father attachment security scores, compared with the control group. The effect sizes were medium-to-large (*d* = .70) for maternal attachment, and very large (*d* = 1.50) for paternal attachment. Such differences suggest a strong discrimination across the clinical and control groups on attachment-related dimensions.

These findings are consistent with the results of earlier studies and provide support for the hypothesis that migraine would be positively associated with anxiety, and negatively associated with attachment security. The magnitude of the differences detected was remarkable, with the majority of effect sizes above the threshold for a medium effect (*d* = .5), and some above the threshold for a large effect (*d =* .8) [27].

### Mediation

Turning to our second aim, we tested a model in which the association between migraine (X) and anxiety (Y) was mediated by attachment (the mediator, M). Fig. 1 depicts a simplified representation of the model Fig. 1. We relied on the SPSS PROCESS macro (Hayes, 2013 – Model #4) to estimate the direct and indirect associations. We controlled for gender and age effects in estimating the model, but such effects are not represented in Fig. 1 for the sake of simplicity.^1^

First we analysed the role of maternal attachment. As for the covariates, anxiety levels were not affected by gender (*t* < 1, *p* > .80), nor by age (β = .12, *p* > .05). Security of maternal attachment was related to gender (β = −.31, *p* < .05), with girls expressing more insecurity about this attachment relationship than boys.

Turning to mediation, the results showed (Fig. 1, upper panel) that migraine was negatively associated with maternal attachment security (β = −.35, *p* < .0001), and in turn maternal attachment security was negatively related to anxiety (β = −.16, *p* < .03). Such pattern yield an estimate of the indirect effect of migraine through maternal attachment of .057, a significant indirect association because the its 95% confidence interval did not include zero (95% CI: .009, .129). Along with the indirect effect through attachment, a direct association of migraine with anxiety was found (β = .32, *p* < .0001).

Descriptively, it was possible to compute how much of the total association between migraine and anxiety followed a direct route, and the proportion of the association that was instead mediated by attachment insecurity. The total effect can be computed by the sum of the direct (.32) and indirect association (.057), and therefore the total effect in this case equaled about .38. Hence, 84% of the total association between migraine and anxiety was attributable to a direct association, and the remaining 16% of the total association was attributable to an indirect path via maternal attachment.

We turned next to the role of paternal attachment (Fig. 1, lower panel)..The mediation model included also the age and gender as covariates (omitted in Fig. 1). Gender did not affect anxiety levels (*t* < 1, *p* > .80), but age was positively associated with anxiety (β = .14, *p* < .04). As found for maternal attachment security, girls expressed more insecurity about the paternal attachment relationship than boys (β = −.13, *p* < .03).

Turning to mediation, migraine was strongly negatively associated with paternal attachment security (β = −.60, *p* < .0001), but paternal attachment security was not associated with anxiety (β = −.09, *t* = 1.08 *p* > .25). As a consequence, the indirect effect of migraine through paternal attachment was not significant (indirect effect = .054, 95% CI: −.041, .164), and only the direct association of migraine with anxiety could be detected in this model (β = .33, *p* < .0001).

### An exploratory model

**Fig. 2:**
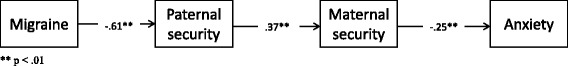
Chain of associations

The two mediation models suggest different conclusions for the role of maternal and paternal attachment. Maternal attachment security was a stronger correlate of anxiety than perceived paternal attachment security, whereas perceived paternal attachment security was more closely associated with migraine. Combining the results of the two models might suggest a more complex pattern of associations, where the association of migraine with anxiety is channeled through two mediators, first paternal attachment and secondly maternal attachment. We investigated thus in a frankly explorative fashion the possibility that migraine would relate with paternal attachment, paternal attachment would then relate to maternal attachment, which in turn would be connected with anxiety (Fig. 2).

We found that migraine was related with paternal attachment (β = −.60, *p* < .001), paternal attachment was connected with maternal attachment (β = .37, *p* < .001), and in turn maternal attachment was associated with anxiety (β = −.25, *p* < .01). This esplorative model might offer a more nuanced interpretation of the processes that may be at play, suggesting avenues for futire research. Insecure maternal attachment appeared to play a pivotal role channeling the association of paternal attachment, and migraine and eventually relating to anxiety.

## Discussion

This study had two objectives. The first was to compare anxiety and perceived attachment security in a group of children and adolescents suffering from MoA and a healthy control group. The second was to test whether the observed group differences in anxiety were mediated by perceived attachment security.

### Between-group differences in anxiety levels and perceived attachment security

With respect to the first objective, our results were in line with most previous studies, confirming that children and adolescents with MoA show higher anxiety levels than their healthy peers [2, 9, 29]. More specifically, our findings replicated previous reports of associations between headache and GAD [30], SAD [31], SA [32–34] and SLA [21, 23] in children and adolescents. Analysis revealed differences between the clinical and control groups with respect to security of attachment to both parental figures. It is well documented that attachment insecurity is associated with several organic conditions of infancy and adolescence, in particular conditions in which emotional triggers have a role, such as headache and abdominal pain [19, 35]. Previous studies [36, 37] have suggested that dysfunctional family relationships are associated with greater pain and disability, in particular migraine [13, 18].

The association between headache and attachment insecurity can be seen as originating from the role of early insecure models for the development and maintenance of migraine symptoms, but also materializing because the enduring stressful condition shapes the child’s perception of availability of attachment figures. We contend that the mediation results we present are consistent with the latter process, which assumes that chronic stress eventually affects attachment beliefs, as discussed below.

### The mediating role of attachment insecurity

As to the second objective of the study, investigating the mediating effect of attachment insecurity on the anxiety symptoms associated with migraine, there were strong negative associations between the paternal and maternal dimensions of attachment security evaluated by the Security Scale and all four forms of anxiety. Our results also indicate that the attachment security dimensions seem to mediate some of the group differences in anxiety. These results provide some support for our mediation hypothesis, which was that the migraine-anxiety association would be at least partially mediated by attachment security. Empirical research has suggested various mechanisms for such a mediation pattern [13]. The role of attachment in increasing anxiety in migraine patients could be attributed to two cumulating sources. On the one hand, insecure attachment developed as a result of the pattern of early interactions might make more severe the child’s anxiety responses to stressful events. Previous studies have evaluated the relationship between attachment styles and pain, showing that perceptions of pain and the related anxious distress might be influenced by attachment models [18, 19]. A recent study found that the anxious-ambivalent attachment style was positively associated with symptoms of anxiety, depression and somatization in children and adolescents diagnosed with migraine [13]. On the other hand, developmental research has demonstrated that stressful events, in turn, can modify extant attachment models. In other words, it seems that, like other organic diseases, headache episodes might represent stressors and thus trigger anxiety responses, which in turn over-activate the attachment system. The tendency to activate the attachment system in order to regulate distress engendered by physical pain is thought to be particularly strong during development. The level of distress young patients experience due to headache episodes is modulated by their perception of the security of their attachment relationships [20, 21]. It should also be noted that children’s lack of trust in parental availability and effectiveness can have a negative impact on the extent to which parental actions modulate their anxiety and distress. The peculiar conditions of headache-related distress may thus activate the attachment system, and the difficulties in meeting the child’s proximity request may bring about an insecure anxious attachment style. A recent study by Barone et al. [38] showed that that migraine attacks weaken children’s attachment security toward their parents, thus undermining the parental confidence in exerting an effective regulatory function on their children’s distress.

The second mediation model evidenced in our results focused on both paternal and maternal attachment and showed that the somatic condition of migraine acted as a stressor, reducing children’s perception of the security of their parental attachments. This result confirms that a state of chronic helplessness and pain constitute a major challenge for the attachment system and family interactions. Interestingly enough, our mediation analysis highlighted differences between the networks of associations involving maternal and paternal attachment. Our clinical group perceived their fathers as much less available and much less emotionally reliable than did the control group, yet such lower paternal attachment security did not have a direct effect on anxiety symptoms. However, paternal attachment insecurity did relate with children’s trust in their mothers, the ultimate buffer of children’s distress, and maternal attachment security in turn was related with anxiety levels. It could be that a child’s loss of trust in his or her father results in a heavy burden being placed on the mother; such burden may become apparent the mother could be thus perceived by the child as being less available and less able to regulate his or her fears and distress, all of which leads to an increase in anxiety symptoms. Recent research [38] highlights how that insecure attachment may hinder parental attempts to reduce children’s distress in the presence of headache attacks.

Overall our results are in keeping with the reported relationship between attachment insecurity and anxiety symptoms, but to the best of our knowledge this is the first study to provide empirical evidence that attachment mediates the association between pediatric headache disorders and anxiety. Our mediation model also suggested that there are differences between the roles played by perceived maternal and paternal attachment security.

### Limitations

We deem the results of this study as potentially fruitful. Of course, further research is needed to allow more detailed conclusions to be drawn. Furthermore, some limitations should be acknowledged. Our sample was relatively modest in size and limited to a specific clinical population, namely children and adolescents with MoA, under observation in a third-level centre, which may increase referral bias. Furthermore, the healthy control group was a convenience sample, and thus subject to the problems inherent in the use of such samples (e.g. relatively high sampling error, selection bias, and other biases). However, the sample size was large enough to guarantee 80% power for the detection of effects in the range suggested by the extant literature.

A second obvious limitation is that our research design cannot be used as the basis for causal inferences. We wish to clarify that the associations and mediation patterns we specified should be considered simply as a summary of a network of associations, and by no means indicative of causal mechanism. The observed network of associations can, nevertheless, be used as the basis for hypotheses about causal relationships that can then be tested using the appropriate experimental designs.

It should also be acknowledged that the measures we used are indicative rather than exhaustive in their respective domains. The Security Scale measures the perceived security of attachment relationships but not attachment style and the SAFA test is a screening instrument rather than a diagnostic instrument. In the future we plan to compare groups of children diagnosed with tension headache, migraine with aura and other chronic conditions. Measures of migraine severity and objective assessments of the psychological traits of clinical subjects and their parents, as well as measures of depression, somatisation and specific phobias would also be useful for characterising parent-child relationships in clinical settings.

## Conclusions

Overall this study provides further confirmation of the established association between MoA diagnosis and anxiety symptoms in childhood and adolescence. Secondly, attachment insecurity can be regarded as one of the factors mediating this association. These conclusions may buttress the importance of individual psychotherapy as a treatment for anxiety in children and adolescents with migraine, as well as highlighting the relevance of psychosocial interventions including family interventions [39].

## Abbreviations

GAD: Generalised anxiety disorder
MoA: Migraine without aura
SA: Scholastic anxiety
SAD: Social anxiety disorder
SAFA: Self Administered Psychiatric Scales for Children and Adolescents
SLA: Separation and loss anxiety

## Footnotes

1 Standard errors of the direct and indirect (mediation) effects were bootstrap-estimated (5000 samples) and 95% confidence intervals were computed to evaluate the reliability of the effects.
